# “Once I take that one bite”: the consideration of harm reduction as a strategy to support dietary change for patients with diabetes

**DOI:** 10.1186/s12902-023-01529-6

**Published:** 2024-01-02

**Authors:** Alexzandra T. Gentsch, Megan K. Reed, Amy Cunningham, Anna Marie Chang, Stephanie Kahn, Danielle Kovalsky, Amanda M. B. Doty, Geoffrey Mills, Judd E. Hollander, Kristin L. Rising

**Affiliations:** 1 Center for Connected Care, Thomas Jefferson University, 1015 Walnut Street, Suite 704, Philadelphia, PA 19107 USA; 2 College of Population Health, Thomas Jefferson University, Philadelphia, PA USA; 3 Department of Family and Community Medicine, Thomas Jefferson University, Philadelphia, USA; 4 Present address: Department of Emergency Medicine, Tower Health Hospital, West Reading, USA

**Keywords:** Diabetes, Harm reduction, Food, Diet

## Abstract

**Background:**

Despite well-established guidelines to treat diabetes, many people with diabetes struggle to manage their disease. For many, this struggle is related to challenges achieving nutrition-related lifestyle changes. We examined how people with diabetes describe barriers to maintaining a healthy diet and considered the benefits of using a harm reduction approach to assist patients to achieve nutrition-related goals.

**Methods:**

This is a secondary analysis of 89 interviews conducted with adults who had type 1 or type 2 diabetes. Interviews were analyzed using a content analysis approach. Themes regarding food or diet were initially captured in a “food” node. Data in the food node were then sub-coded for this analysis, again using a content analysis approach.

**Results:**

Participants frequently used addiction language to talk about their relationship with food, at times referring to themselves as “an addict” and describing food as “their drug.” Participants perceived their unhealthy food choices either as a sign of weakness or as “cheating.” They also identified food’s ability to comfort them and an unwillingness to change as particular challenges to sustaining a healthier diet.

**Conclusion:**

Participants often described their relationship with food through an addiction lens. A harm reduction approach has been associated with positive outcomes among those with substance abuse disorder. Patient-centered communication incorporating the harm reduction model may improve the patient-clinician relationship and thus improve patient outcomes and quality-of-life while reducing health-related stigma in diabetes care. Future work should explore the effectiveness of this approach in patients with diabetes.

**Trial registration:**

Registered on ClinicalTrials.gov, NCT02792777. Registration information submitted 02/06/2016, with the registration first posted on the ClinicalTrials.gov website 08/06/2016. Data collection began on 29/04/2016.

## Background

In 2020, the Centers for Disease Control reported 34.2 million Americans, 10.5% of the population, have diabetes mellitus and 88 million (34.5%) have prediabetes [1]. The American Diabetes Association reported that the total cost to treat diabetes was $327 billion, a 26% increase from 2012 to 2017 [2]. Despite existence of evidence-based treatments for diabetes, many people continue to struggle with diabetes management, with diet-related issues reported as one of the most prevalent difficulties related to diabetes management [3–6].

Recommendations for people with diabetes to adopt healthier lifestyles, particularly around diet, are predicated on the assumption that people have the ability and freedom to make “healthier” choices. Furthermore, these recommendations often assume that everyone is in the same state of readiness to make substantial sustained lifestyle changes. Literature suggests many reasons that patients with diabetes have difficulty adhering to treatment plans regarding their diet including self-discipline, deficit of knowledge (i.e. understanding what they should eat, why, and how), coping with everyday stress, negotiating with family members, and managing the social significance of food [5, 7, 8]. A systematic review of randomized controlled trials conducted from 1975 to 2015 that looked at interventions used to change diet and physical behaviors of people with type 2 diabetes concluded clinically significant changes could be attained for up to 6 months, but they were not sustained at 12 and 24 months [9]. While underlying biological causes of obesity and appetite regulation certainly impact individuals’ diabetes outcomes, this review identified other important factors that influence participants’ ability to improve or sustain hemoglobin A1c (HbA1c) levels including attempts to change multiple behaviors simultaneously versus changing behaviors individually, the sequential order interventions were attempted, and a consideration of which interventions were uniquely best suited for an individual [9]. These studies suggest that further work is needed to develop patient-centered approaches to enable more sustained dietary change.

One such approach to center the wishes, motivation, and ability of a client at a current point in time is harm reduction. Harm reduction is a non-judgmental approach, often applied to substance use, that recognizes behavior change, often happens in increments and meets people “where they’re at” [10]. Evidence over decades demonstrates how people who access harm reduction programs have lower rates of HIV, are less likely to share syringes, and are more likely to decrease their drug use overall [11]. Listening directly to the words used by patients with diabetes may indicate possible clinical applications of a harm reduction approach. Harm reduction as applied to diabetes could encompass a range of behaviors related to diet, self-testing blood glucose levels, medication adherence, and other such self-management behaviors related to diabetes.

The goal of this work is to examine how people with type 1 and type 2 diabetes describe their relationship with food and their barriers to achieving and maintaining a healthy diet. This work considers the potential benefits of harm reduction to assist clinicians in developing individualized and more successful treatment plans.

## Methods

### Research design

This is a secondary analysis of individual qualitative interview data from a parent study that compared the comprehensiveness and efficiency of semi-structured interviews to group concept mapping for eliciting patient-important outcomes for diabetes care. The full methods of this IRB approved parent study have been described elsewhere [12]. The aim of this study is to identify how data from the parent study informs the benefit of a harm reduction model for patients with diabetes. All methods were carried out in accordance with relevant guidelines and regulations.

### Sample

Eligible individuals were recruited from an urban academic medical center in Philadelphia, PA. English-speaking adults (age 18 and older) with type 1 or type 2 diabetes who were able to provide informed consent were included. Patients were recruited: 1) during an emergency department (ED) visit (acute care), 2) within 7 days post-hospital discharge (post-acute care), and 3) at the time of a scheduled primary care visit (primary care). All participants had uncontrolled diabetes defined as follows: presented to the ED with a diabetes-related problem (acute care), admitted to the hospital for a diabetes-related problem (post-acute care), or had at least 2 measurements of HbA1c > 7.5% in the prior year (primary care).

Exclusion criteria included patients with a new diagnosis of diabetes during index visit; having a significant permanent complication related to diabetes, including end stage renal disease, amputation, or blindness; undergoing medical clearance for a detox center or any involuntary court or magistrate order; in police custody or currently incarcerated; or having major communication barriers such as visual or hearing impairment or dementia that would compromise ability to give written informed consent.

The entire study was conducted in close collaboration with the Patient and Key Stakeholder Advisory Board (PAKSAB). The PAKSAB is an advisory board including patient advocates experienced with helping chronically disabled and disadvantaged populations negotiate various healthcare settings as well as key representatives from diverse domains of the care pathway. Two members of the PAKSAB worked as members of the research team for this project, and were involved in all aspects of interview development, conduct, and analysis.

### Data collection

Patients recruited from the acute care group were identified and screened using the electronic medical record (EMR) and were approached during their ED visit. Patients recruited from the post-acute and primary care group were identified and screened using auto-generated lists from the inpatient and outpatient EMR. Post-acute patients were contacted, consented and interviewed by telephone. Primary care patients were contacted by phone prior to a scheduled visit to assess interest in study participation and were subsequently consented and interviewed on-site immediately before or after their primary care appointment. All participants provided informed consent. All participants were compensated $25. Research approval was obtained from Thomas Jefferson University’s Institutional Review Board.

An open-ended, semi-structured interview guide was used to discuss outcomes most important to participants when making decisions regarding the management of their diabetes. The research team collaborated with the PAKSAB to develop, test, and refine the guide. Interview questions asked about participants’ beliefs regarding the cause of their diabetes, their challenges and worries caring for their disease, and what their care goals were.

Two PAKSAB members and two research team members conducted interviews that were audio recorded and lasted approximately 30 min. Demographic information was collected at the end of each interview and a medical chart review was completed to collect the most recent recorded HbA1c and body mass index (BMI). Interviews were conducted separately in each of the three groups until thematic saturation was reached. Thus, the team aimed for approximately 90 interviews, with 30 in each group, and the final number determined using thematic saturation during analysis [12].

### Analyses

Audio-recorded interviews were transcribed professionally with identifying information removed. Transcripts were checked by a team member for accuracy and imported into NVivo 11.0 [13]. Three team members served as coders. One third of all interviews were coded by all three coders to ensure consistency of coding and check for coder drift, with the remaining transcripts coded by one of the three coders. A fourth team member experienced in qualitative methodology oversaw the process and regularly reviewed Kappa coefficients and percentage agreement to assess interrater reliability.

Interview analysis for the parent study was conducted with a conventional content analysis approach [14]. The coders developed the codebook by immersing themselves in the transcripts, identifying themes that emerged, and coding transcripts independently. They then met to review coding, resolve discrepancies, and refine the codebook iteratively. Two PAKSAB members and one research team member served as coders, and the full PAKSAB reviewed and discussed interview findings with the research team. A comparison of themes that emerged across patient settings (e.g. acute care vs. post-acute care) was also initially conducted and no significant differences were identified [12].

Any themes about food or diet were initially captured in a “food” node. For the purposes of this secondary analysis, the research team then sub-coded all data in the food node, again using a content analysis approach. This coding was conducted by two research team members not involved in initial analysis. Coders met regularly to review coding and resolve discrepancies based on Kappa coefficients and percentage agreement. This manuscript discusses the themes that emerged under the broad category of challenges to achieve diet goals from this secondary analysis of data in the food node. Code definitions, sub codes identified within these two categories and the number of times codes were referenced can be found in Table 1.

**Table 1:** Codebook for nodes related to food challenges, including frequency of codes

Node	Definition	# of interviews coded to a node	# quotes coded to a node
Challenges to achieve diet goals	Challenges to achieve diet goals or inability to translate knowledge to practice	75	475
Access	Not having access to foods participants think they should be eating.	11	35
Availability of unhealthy food	References to the availability of unhealthy food	4	9
Cheating, weakness, addiction	References to cheating ,weakness, or addiction to food (and any related stem words)	18	57
Comforted by food when stressed	References to food when a participant describes feeling stressed	12	39
Eating out	Includes any challenging aspects of eating out	3	5
Education and guidance	Participants desire for more guidance or education around nutrition	10	28
Learned lifestyle	Lifestyles participants learned that made it difficult to eat healthier	11	16
Social pressures	Any references to social pressures	13	31
Time concerns	How time restraints affect diet	7	19
Unwillingness, resistance, apathy to change	Unwillingness, resistance, or apathy to change	20	33
Other challenges	Any other challenges to achieve diet goals or inability to translate knowledge to practice	21	45

## Results

Ninety-five individuals were enrolled and 89 interviews were analyzed in this work (30 participants receiving acute care, 29 receiving post-acute care, and 30 receiving primary care participants); two participants could not complete their interviews, two were ultimately determined to meet exclusion criteria, and two recordings were not usable. The mean participant age was 55 years, and the majority of participants were black (68%), female (55%), high-school graduates (68%), and reported having a diagnosis of diabetes for over five years (83%). The mean HbA1c was 10.2% (SD 3.3) (Table 2).

**Table 2:** Participant demographics (*N* = 89)

Characteristic	n (%)
Age – Mean (SD)	54.6 (13.8)
Ethnicity	
Hispanic/Latino	8 (9)
Not Hispanic/Latino	80 (90)
Race	
White	24 (27)
Black	60 (68)
Other	4 (5)
Sex	
Male	40 (45)
Female	49 (55)
HbA1c % – mean (SD)	10.2 (3.3)
Body Mass Index – kg/m^2^ (SD)	34.8 (10.3)
Hospital admits in past 12 months – mean (SD)	2.3(4.1)
ED visits in past 12 months – mean (SD)	2.8(4.3)
Doctor visits in past 12 months – mean (SD)	11.2(4.3)
Education	
Less than high school	4 (5)
High school graduate	68 (76)
College degree	4 (5)
Post-grad degree	13 (15)
Income	
<$10,000	15 (21)
$10,000-$24,999	22 (31)
$25,000-$49,999	19 (27)
$50,000-$99,999	7 (10)
>=$100,000	8 (11)
Years since diabetes diagnosis	
< 1 year	2 (2)
1–5 years	12 (13)
> 5 years	74 (83)
Type of diabetes	
Type 1 Diabetes	4 (5)
Type 2 Diabetes	82 (92)
Unknown	3 (3)
Health status, assessed with the question: “In general, would you say your health is…” – mean (SD) (range 1–5: 1 = excellent, 2 = very good, 3 = good, 4 = fair, 5 = poor)	3.6 (0.9)

Many participants discussed barriers to healthy eating that have already been cited in the literature (e.g., social pressures, limited access to healthy foods, learned unhealthy eating habits, and lack of time to meal prep), and thus these barriers are not the focus of analysis. How participants otherwise talked about food fell into two categories: 1) challenges to achieve diet goals and 2) approaches to changing diet. When describing challenges to achieve diet goals participants talked about addiction, cheating, feeling comforted by food, and an unwillingness to change. We discuss these four primary challenges in depth below.

### Addiction

Participants frequently used addiction language to talk about their relationship with food. Many described how food felt like “their drug”, and some referred to themselves explicitly as “addicts”. Participants linked their relationship with food to their personal experiences with a substance use disorder. As one participant stated,

“*It’s hard, because I’m in recovery. I have 25 years clean. So I don’t smoke. I don’t drink. I don’t do any of those things. So food was what I had left, and now unfortunately I don’t have that anymore. So it’s kind of frustrating. That was like my comfort thing.*” (ID 110)

Another participant included food alongside substances as a maladaptive form of solace when struggling with difficult emotions. He shared,

“*If something was bothering me, whatever, I wouldn’t talk about it and internalize it. And over the period of time it builds up, builds up, builds up until I’m exploded with it. And I would do damage to myself. I would never hurt nobody else. It was just that I would do things to hurt myself. Drugs, alcohol, food, whatever.*” (ID 217)

Participants also used expressions typically associated with substance use recovery like “pull off the highway,” “going rogue,” and “fell off the wagon” to convey moments when they deviated from their recommend diet. When asked what one participant meant when she mentioned that she “falls off the wagon,” she replied,

“*Well, I have – well, I’ll say this. I’ll say that I’m not going to meet the group for happy hour. I’m not gonna do that. And I will stick to that for two, three, four days, and fall off the wagon.*” (ID 130)

Many participants described how they wanted to eat healthier but struggled to do so because of triggers. One participant explained,

“*[My doctor] told me to stay away from what I like to eat. Just stay away from it, drink plenty of water, cranberry juice. Eat a lot of fruit and salad. And I just – but that ain’t working for me. I tried. Yeah. But going past the bakeries and smelling that good bakery, and that’s messed everything up.*” (ID 209)

### Cheating

Participants described indulging in unhealthy foods either as a sign of weakness or as “cheating.” One participant stated,

*“I love sweets – cookies, ice cream. I try to cut it out now. I still cheat. Pizza [chuckles]. Pizza is ridiculous, and that’s really bad for you. They say that’s like a poison.” (ID 227)*

While some patients acknowledged “cheating,” “a cheat day,” or said “I cheat,” some circled back to an addiction comparison. One participant reflected on their relationship with food and shared,

“*I can do good for a while, and then I just feel like I’m just fed up, I’m gonna cheat a little bit. And unfortunately my cheating isn’t for a little bit. I can go back to my old routines and then I have to struggle to get back to where I was. Just like I always keep in mind about an alcoholic – about how once they slip, they – it’s a struggle for them to get back on board.*” (ID 119)

Another participant described her struggle with ice cream saying,

“*Once I do something like – I like ice cream. But I can’t take that one bite. And so once I take that one bite, it’s like I’m gonna cheat today. But then I know cheating today – saying I’m gonna cheat today and not eat it for a month ain’t helping me because I just ate a half-gallon of ice cream.*” (ID 103)

### Comforted by food

Participants also talked about unhealthy foods and overeating as a way to cope with challenging emotions. One participant stated,

“*If I got upset, well, the first thing I do is eat. I go straight to food. And I know that’s not right*.” (ID 105)

Another noted,

“*Normally if I’m not eating the way I’m supposed to, it’s because I’m just having a really bad day. And I’ll cheat and have a half a candy bar or some M&Ms.*” (ID 110)

Some participants described how it was helpful to cope with moments of sadness or periods of grief. One participant offered a recent example saying,

“*And like my chocolate. I know it’s not good. But when like things happen in life, like I just lost my best friend, and I had some chocolate to soothe my grief.*” (ID 111)

Other participants made sense of their situation by labeling themselves an “emotional eater” or a “depression eater.” While many described how comforting food could be, one participant literally referred to food as her “companion.” (ID 119) Participant ID 124 described how a combination of challenging physical and emotional circumstances resulted in her sub optimal diet:

“*I’m going to be honest with you. I’ve been cheating like hell. And I don’t – after I got sick, I’ve been on my Ps and Qs. But when you’re depressed and you’re tired of coming to the doctors and you’re out all the time in the streets, you pick up whatever you can find to eat.*”

### Unwillingness to change

Finally, some participants reported having conversations with their clinician about their diet and then choosing to disregard clinician recommendations. They described being unwilling or feeling unable to change. Participant ID 109 described his situation simply as a “conflict” as he explained they he hated soda, yet he continued to drink it:

“*The struggles that I face sometimes where I have to decide whether I want that cake or the peanuts or the soda. Which I hate soda now so much but I still like to drink it. But it’s a conflict there. And I talk to my psychiatrist about why I’m having this conflict.*” (ID 109)

Participants showed varying levels of remorse when discussing their unwillingness to change; some expressed guilt for not being able to make these changes whereas others were unapologetic in their actions. One participant described their disappointment saying,

“*you throw all logic out the window and say, oh my God, it looks so good. I have to eat that. And you eat it and you then – you have eater’s regret afterward.*” (ID 114)

Other participants, like ID 218, described how they chose to eat foods that they knew would make them feel unwell:

“*I’ve been trying to manage it. But I’ve been sneaking food. I’ve been sneaking stuff… Sometimes I cheat and I go get me some real candy. And I’m not supposed to eat it, but I do. And after I eat it, I get lightheaded. So I’ll be trying not to do it, but I do. I do.*”

Another recognized her behavior was problematic and described difficulty facing the implications, saying,

“*There’s a major struggle between me and ice cream and my diabetes. I say, well if it hurts me, it hurts me. But in the long run, I know it will hurt me, so I’m struggling with that right now.*” (ID 226)

While some participants described feeling guilty about their diet or a struggle to eat more healthy, others showed no remorse, as demonstrated by a participant who said

“*when they find out that I have diabetes, they was like well you know you need to eat more healthier. And I said, guess what? I’m gonna eat what I’m gonna eat and when I die I’m gonna die*.” (ID 102)

Participant ID 120, described a sense of ambivalence when he shared,

“*All my doctors said, [Participant 120], you gotta lose weight, you gotta lose weight, you gotta lose weight. What am I doing? Nothing. I like eating*.”

## Discussion

Among this large sample of participants with uncontrolled diabetes, many participants discussed the negative impact of their relationships with food and their ability to achieve healthy diets. They described relationships that included addiction, cheating, the need for comfort and unwillingness to change, and routinely used terminology generally associated with, and assigned to, people who use drugs (e.g., “cheat,” “slip,” “old routines”).

People eat food and use substances in part because of the pleasure-inducing effects on the brain [15, 16]. Just as people with addiction experience public stigma, so do people with diabetes, especially those with higher BMI [17, 18]. People who experience public stigma often internalize stigma, leading to an exacerbation of negative behavior. Research demonstrates that stigma toward people who use drugs exacerbates the negative impacts of drug use [19, 20]. For patients with diabetes, this may manifest by eating foods that negatively impact diabetes outcomes in terms of treatment adherence [21].

A review of the experiences and perceptions of people with type 1 and type 2 diabetes demonstrated that individuals report stigma as a significant concern impacting both their professional and personal lives [22]. Participants across several studies expressed shame, embarrassment, and feelings of failure, especially those with type 2 diabetes. Further, participants reported that the extent of shame and stigma people with diabetes experience can be so immense that they are willing to compromise efforts to maintain a healthy diet in an attempt to conceal their health condition [22]. Importantly, research also indicates that clinicians may worsen internalized stigma in patients and, therefore, undermine their diabetes management [23, 24]. Furthermore, when someone is seen as the whole of a singular behavior (e.g., an “addict” for someone who uses drugs), they risk feeling shame globally, which is associated with greater substance use [25]. Shame, in turn, can lead to hiding behaviors and less self-disclosure in the clinical relationship [26]. Recognizing the agency of the patient – whether using substances or engaging in clinically-damaging eating behavior – can avoid or counteract these feelings of shame. For these reasons, stigma-free, patient-centered, and person-first language are recommended by the American Diabetes Association [24].

Based on our findings linking food to addiction, our data suggest that a harm reduction approach may be beneficial for patients with diabetes who are struggling to improve their diet. Harm reduction is an evidence-based strategy that recognizes that behavior change occurs along a continuum, often improving incrementally [27, 28]. Principles of harm reduction include humanism, pragmatism, individualism, autonomy, incrementalism, and accountability without termination (Table 3). Harm reduction is typically applied to drug addiction and recognizes the dignity and autonomy of people who use drugs [29]. The goal of harm reduction is not always abstinence, but positive change for the individual [10]. Harm reduction has also been applied philosophically or practically (e.g., through interventions) to disordered eating [30], smoking [31], and medication adherence for mental illness [32]. Based on the themes identified in our qualitative study, a similar approach aimed at establishing patient-centered goals and fostering small, incremental changes in controlling diabetes without judgment could be successful and should be the focus of future studies.

**Table 3:** Harm reduction principles applied to diabetes patient-clinician interactions

Principle^a^	Definition
Humanism	Respect for the dignity of the patient; recognition that patients engage in behaviors for some benefit to themselves
Pragmatism	The expectation that patients will behave perfectly at all times is unrealistic; upstream factors outside of patient control also drives behavior
Individualism	Patients have their own unique needs and capabilities; patients need different treatment plans from one another
Autonomy	Clinicians can guide choices but the decision is ultimately made by the patient
Incrementalism	Positive change happens in changes and often takes years; return to previous behavior is normal
Accountability without termination	Clinicians explain consequences of choices to patients without judgement, patients decide accordingly; clinicians are never punitive

^a^ Principles adapted from Hawk et al. 2017 [33]

Recent critiques of harm reduction in addiction management assert that these principles must extend beyond the individual level, or they risk shifting blame for behavior back onto individuals, even in situations where structural factors make behavioral change impossible [34]. Those with diabetes who have food insecurity have more limited means to control their diets than others. Care must be taken by clinicians when working with patients to recognize which factors are modifiable by patients and which rely on upstream solutions. As a longer term strategy, harm reduction-focused clinicians should focus on the structural conditions that facilitate diabetes as well as addressing individual choices.

### Limitations

Our sample consisted of an urban, primarily Black population with uncontrolled diabetes. This may limit the transferability of findings to other groups and populations. Eligible individuals were also a convenience sample of those who were able to be contacted and agreed to participate, which may contribute to selection bias. This sample was also primarily diagnosed with type 2 diabetes, therefore further research may be needed to probe people diagnosed with type 1 diabetes. In addition, this was a secondary analysis of an existing data set, with the primary study question focused on patients’ goals related to seeking diabetes treatment. The theme of challenges related to food and addiction emerged during analysis, and the interview guide was not designed to probe for in-depth discussions of how a harm reduction approach could benefit individual patients. Despite these limitations, our study was conducted across a large sample of participants and identified salient themes related to food and addiction. Future studies are needed to explore this topic in greater depth.

## Conclusion

In this sample of patients with diabetes, many framed their relationship with food and related struggles with improving their diet in an addiction lens. Harm reduction-focused interventions have been associated with positive changes for those with substance use disorders and offer potential benefit for patients with diabetes who struggle to adequately control their diet.

Harm reduction is increasingly being conceptualized as applicable to a variety of chronic health conditions, including for disordered eating, but has not yet appeared in literature to inform diabetes management [30, 35, 36]. Patient-centered communication is central in the harm reduction model. Some patients in our study disregarded clinician instructions (e.g., to “stay away” from certain foods entirely), which is indicative that diet plans were not feasible for patients. Other patients expressed shame over their diets rather than feeling empowered by changes they had made (e.g., eating pizza less frequently throughout the week). Improved communication using these principles may improve the patient-clinician relationship, which is an important moderator of improved diabetes outcomes [37, 38]. Conversations with patients about their diets can inform the extent to which certain behaviors are attributable to cultural norms, food insecurity, or long-term habits. Language around diet and setbacks should be normalized rather than framed as “cheating” [38]. This non-judgmental approach and bidirectional sharing of information will build rapport and make conversations about goal-setting realistic and tailored to the patient’s abilities and wishes. Practitioners should recognize that traditional benchmarks of “success” and “adherence” may need to be adjusted accordingly. For patients who consistently struggle with diet and exercise changes, doctors may explore pursuing a treatment-focused approach without behavioral changes. This shift in focus may improve patient outcomes and quality-of-life while reducing health-related stigma in diabetes care. Future work is needed to further explore optimal design and ultimate impact of harm-reduction strategies focused on diet management for patients with diabetes.

## Data Availability

The datasets used and/or analyzed during the current study are available from the corresponding author on reasonable request.

## Abbreviations

HbA1c: Hemoglobin A1c
EMR: Electronic medical record
ED: Emergency department
PAKSAB: Patient and Key Stakeholder Advisory Board
BMI: Body mass index
